# Exploratory Monitoring of the Quality and Authenticity of Commercial Honey in Ecuador

**DOI:** 10.3390/foods8030105

**Published:** 2019-03-20

**Authors:** Lorena Salvador, Michelle Guijarro, Daniela Rubio, Bolívar Aucatoma, Tanya Guillén, Paul Vargas Jentzsch, Valerian Ciobotă, Linda Stolker, Sonia Ulic, Luis Vásquez, Patricia Garrido, Juan Bravo, Luis Ramos Guerrero

**Affiliations:** 1 Centro de Investigación de Alimentos, CIAL, Universidad UTE, EC170527 Quito, Ecuador; lorestefania19@hotmail.com (L.S.); mguijarro@ute.edu.ec (M.G.); dannarubio@gmail.com (D.R.); patricia.garrido@ute.edu.ec (P.G.); juan.bravo@ute.edu.ec (J.B.); 2 Centro de Investigación de la Caña de Azúcar, CINCAE, El Triunfo 091601, Ecuador; baucatoma@cincae.org (B.A.); tguillen@cincae.org (T.G.); 3 Departamento de Ciencias Nucleares, Facultad de Ingeniería Química y Agroindustria, Escuela Politécnica Nacional, Quito EC170525, Ecuador; paul_5151@yahoo.com; 4 Rigaku Analytical Devices, Inc., Wilmington, NC 01887, USA; Valerian.Ciobota@rigaku.com; 5 Wageningen University & Research Akkermaalsbos 2, 6708 WB Wageningen, The Netherlands; linda.stolker@wur.nl; 6 CEQUINOR (UNLP-CONICET), Universidad Nacional de La Plata, La Plata 1900, Argentina; sonia@quimica.unlp.edu.ar; 7 Facultad de Ciencias de la Seguridad y Gestión de Riesgos, Universidad Internacional del Ecuador, Quito EC170504, Ecuador; lvasquez@uide.edu.ec

**Keywords:** Honey, adulteration, Raman spectroscopy, infrared spectroscopy, chemometric

## Abstract

Honey is one of the oldest sweetening foods and has economic importance, making this product attractive to adulteration with cheap sugars. This can cause a critical problem in the honey industry and a possible health risk. The present work has the aim of evaluating the authenticity of honey commercialized in two different provinces of Ecuador (Pichincha and Loja) by performing physicochemical and spectroscopic analyses. For this study 25 samples were collected from different places and markets and characterized by water, sucrose, reducing sugars and electric conductivity measurement. Also, their Raman and Infrared (IR) spectra were recorded and analysed using a Principal Component Analysis (PCA) in order to verify the quality of the honeys. In addition, a screening of several pesticides was performed in order to verify possible chemical threats to human health and honey bees. It was found that 8 samples have a deviation from the Standard established parameters. Two of them have a high difference in the content of sucrose and reducing sugars, which are located deviated from all the other samples in the PCA of the applied vibrational spectroscopy (IR/Raman), shaping two clear clusters. The results show that Raman and IR spectroscopy is appropriate techniques for the quality control of honey and correlates well with the physicochemical analyses.

## 1. Introduction

Honey is produced by honey bees (mainly *Apis mellifera*) and is considered a valuable food commodity due to its good taste, nutrients, availability and health-giving properties [1]. There are several reports of ancient uses of honey, not only as food but also for medicinal purposes [2]. Moreover, numerous recent studies reported that honey possess antimicrobial, antioxidant and anti-inflammatory properties and its use as an antiviral, antiparasitic, antimutagenic and anticancer agent was suggested [3,4,5]. Honey is composed mostly of sugars, mainly fructose and glucose and other constituents in a smaller proportion such as enzymes, amino acids, organic acids, carotenoids, vitamins, minerals, aromatic substances and phenolic compounds [6,7], which are substances responsible for the properties mentioned above.

Taking into account the nutritional and therapeutic importance of honey and its high commercial value, the occurrence in the market of adulterated products cannot be ruled out. Common adulteration methods of honey include the addition of sugar syrups with a lower economic value and similar composition (e.g., corn syrup with high fructose content, maltose syrup, inverted syrups and others) and low-quality honeys [8,9]. First reports on honey adulteration date back to the 1970s and since that time the number of cases increased significantly, thus affecting the different honest producers and consumers [10,11,12].

The quality of honey depends on its components. According to the Codex Alimentarius and the Ecuadorian Technical Standard NTE INEN 1572, honey must not have added any additional ingredients as well as foreign matters, aroma, taste or pigments derived from the processing or storage, otherwise, its authenticity might be questioned. Additional parameters to assess the authenticity of honey and evaluate its overall quality include electrical conductivity, moisture, sugar (fructose, glucose, sucrose), hydroxymethylfurfural contents, among others [13,14].

The adulteration of honey is not a geographically isolated problem, in fact, it has worldwide implications. Several publications on instrumental techniques for the detection of adulterated honey can be found in scientific literature; the detection and discrimination of unifloral and multifloral honeys, as well as those adulterated honeys, was possible principally by means of Nuclear Magnetic Resonance (NMR), infrared spectroscopy and liquid chromatography, among others [15,16,17,18,19,20,21,22], usually also combined with the application of chemometric methods. More recently, the use of artificial senses emerged as a tool to properly evaluate the authenticity of honey [23,24,25].

In Ecuador, fake honeys were detected by mean of NMR spectroscopy in three different provinces [26]. Other cases have been reported in Brazil and Costa Rica [27,28], where analysis confirmed mainly the addition of artificial syrups. Therefore, there are reasons to suspect that the adulteration and even the counterfeit of honeys are still taking place in Ecuador and other countries in the region. It is crucial to provide to the control institutions appropriate control methods based on agile techniques, therefore the use of portable and/or handheld devices is highly recommended for in situ measurements (when possible). In recent years, vibrational spectroscopic has become a powerful tool for the detection of adulterations. The use of handheld Raman devices for the identification of adulterated essential oils, counterfeit stevia and quality control of agricultural products (e.g., cocoa beans) was successfully carried out in the past [29,30,31,32] and this offers new opportunities for analysis in different fields.

On the other hand, bees have an essential job as pollinators which has been affected due to the excessive use of pesticides. Pesticides are present in almost all crops, so they could directly affect the beekeeping products (honey bees, honey, pollen, beeswax). Its use has negative effects in food safety of honey but also for bees, causing problems like feeding, learning and memory performance, decrease in population and reproduction [33,34]. The European Union and the Codex Alimentarius have regulated the maximum residue limits (MRLs) for pesticides in plant products and other matrices such as honey. Different kind of pesticides were detected in honey such as carbamates, organophosphates, pyrethroids, formamidines [35], however, recently neonicotinoids have attracted attention because its widespread use and its persistence in the environment, making easier their contact with bees [36]. In fact, evidence linking the use of neonicotinoids and a decline in bee health was published recently [37].

In this work, the quality of 25 commercial honeys from two Ecuadorian provinces was evaluated. Basic characterization of honeys was carried out and these results were complemented with Raman and infrared measurements. The spectroscopic data were processed with chemometric methods. In addition, a pesticides screening of the samples was performed in order to verify possible chemical threats to human health and honey bees.

## 2. Materials and Methods 

### 2.1. Honey Samples

A total of 25 commercial samples of honey obtained from the provinces of Pichincha (15) and Loja (10), were evaluated. The samples come from supermarkets, health food stores, farms and popular markets. Most of the samples do not declare in the label the origin of the honey (only in one sample, it corresponds to local and imported honey). Regarding to the botanical origin of the product it is indicated that five samples corresponds to a multifloral origin and four mainly a mixture from eucalyptus, clover, alfalfa and avocado. According to the governmental agency for agriculture and livestock from Ecuador (AGROCALIDAD), *Apis mellifera* is the main species of bee related to honey production in Ecuador.

### 2.2. Basic Characterization of Honey Samples

Percentage of glucose, fructose and sucrose, moisture content and electrical conductivity were determined using the methods mentioned in the Ecuadorian Technical Standard NTE INEN 1572 [14]. The separation and quantification of sucrose, glucose and fructose was carried out using a Liquid Chromatography (Agilent Technologies 1260 Infinity II) with a manual injector 61328C (1260 Man. Inj.), a quaternary pump 67111A (1260 Quat Pump Vl), a multicolumn furnace 67116A (1260 MCT) and a refractive index detector 67162A (1260 RID). For the separation, a Hi-Plex Ca column of 300 × 7.7 mm was used together with a mobile phase of deionized water (conductivity < 1 µS/cm). The flow rate of the mobile phase was 0.700 mL/min and a temperature of 80 °C was set for the elution. An amount of 1.000 ± 0.001 g of the sample was placed into a glass vessel and dissolved with enough deionized water to obtain 100 g of solution. The solution was filtered through a regenerated cellulose syringe filters (0.20 µm pore size and 25 mm diameter) and collected in a 2-mL vial. Afterward, 20 µL of the solution was injected into the HPLC. For quantification purposes, a calibration curve was prepared with a mix of three external standards of sucrose, glucose and fructose (Merck, 99%) in the range of 0.10–0.20% *w*/*w* for sucrose and 0.10–0.40% *w*/*w* for glucose and fructose.

The moisture determination was carried out by gravimetric methods (NTE INEN 0265 Standard) as follows: 6.000 ± 0.001 g of sample was dried in an oven (Memmert) at 65 °C for 24 hours, then cooled to room temperature into a desiccator and weighed. The moisture content was expressed as percentage.

For the electrical conductivity measurements, the samples were homogenized by heating them up to 40 °C, then weighted 2.000 ± 0.001 g of anhydrous honey and dissolved with purified water type I, leading to a final volume of 10 mL. The electrical conductivity of the solution was evaluated at 20 °C ± 2 °C using a Thermo Scientific conductivity meter [14].

### 2.3. Raman and Infrared Spectroscopy

Raman measurements were performed using a handheld Raman, Progeny^TM^ (Rigaku Analytical Devices, Wilmington, MA, USA), spectrometer equipped with a 1064-nm Nd:YAG laser and a Peltier cooled InGaAs detector. The total laser exposure time for each Raman measurement, performed through transparent glass vials, was 6 s with a laser power of 490 mW. The accuracy and precision of the Raman bands of the acquired spectra were below 3 cm^−1^ and 1.5 cm^−1^, respectively. Ten measurements per day were performed on each sample and each sample was measured three times on three different days, in order to consider possible day-to-day variations. 

Infrared spectra were recorded using an Agilent Cary 630 FTIR spectrometer with an ATR device. For each measurement, 128 scans were performed at a resolution of 2 cm^−1^ in a spectral range of 4000 to 600 cm^−1^. After each measurement, the sample residues were removed from the diamond tip and cleaned with acetone. The samples were taken from three different points of the container and four spectra were obtained from each point. The spectroscopic data were used to calculate the average spectra for each sample and perform the multivariate analysis. 

### 2.4. Multivariate Analysis

The Raman and IR spectroscopic data were processed using the “R” software [38]. The data pre-processing included baseline correction and normalization. The background of the IR spectra was removed by using a statistics-sensitive non-linear iterative peak-clipping algorithm (SNIP) [39] with a fourth-order clipping filter. The background of the Raman spectra was automatically removed after each data collection by the handheld Raman device, using its proprietary baseline correction algorithm. Subsequently, all the spectra were min-max normalized. A principal component analysis (PCA) was performed to examine the similarities and differences of the Raman and IR spectra.

### 2.5. Pesticides Residues

Thirty-four pesticides and/or pesticide metabolites were determined in honey samples (Table S2). They were determined by their extraction from 1.0000 ± 0.0001 g of honey and the subsequent determination by Liquid Chromatography. The extraction of the pesticides was performed by the QuEChERS method and then the extracts were analysed by Liquid Chromatography coupled to Tandem Mass Spectrometry (LC-MS/MS). The LC-MS system consisted of an Ultra-Fast Liquid Chromatograph (UFLC XR, Shimadzu) and a QTRAP 5500 MS (Applied Biosystems). MS conditions were as follows: scan type MRM (multi reaction monitoring); ion source ESI(-) and ESI(+); resolution Q1 an Q3 in unit; setting time 5 msec; MR pause 5 msec; curtain gas (CUR) 20; collision gas (CAD) medium; temperature (TEM)) 300 Celsius degrees. Ion source gas 1 and gas 2, both 60; ionspray voltage (IS) 5500 V (ESI +) and -4000 (ESI-); entrance potential (EP) 10 (ESI+) and -10 (ESI-) [40]. A Restek Ultra Aqueous C18 (100 mm × 2.1 mm, 3.0 μm) column was used and set at a temperature of 40 °C. The step gradient (solvent A, 5 mM ammonium formate + 0.1% formic acid in Milli-Q water; solvent B, 5 mM ammonium formate + 0.1% formic acid in methanol/Milli-Q water 95/5 (*v*/*v*)) was 0–5 min linear increase to 50% B (initially 100% A), 5-6 min linear increase to 100% B, which was held for 2 min. The injection volume was 10 μL, the flow rate of the mobile phase was 0.400 mL/min. Concentrations of standard solutions were corrected for purity. For quantification purposes, a calibration curve was prepared with a mix external standards of the selected pesticides. In the LC-MS/MS sequence, five sample extracts were bracketed by a matrix-matched calibration standard. The concentration in the sample was calculated by comparison of the average area of the matrix-matched calibration standard and the peak area of the specific pesticide in the sample.

## 3. Results and Discussion

### 3.1. Basic Characterization of Honey Samples 

**Figure 1:**
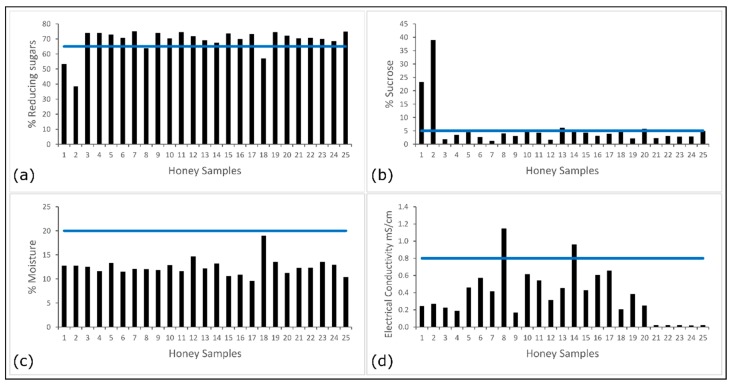
Results of basic characterization of honey samples: (**a**) Content of reducing sugars (%), (**b**) Content of sucrose (%), (**c**) Moisture content (%) and (**d**) Electrical conductivity (mS/cm). The solid lines in the graphs indicate the limit for each parameter that allows evaluating the authenticity of honey.

The results of the basic characterization of honey samples are detailed in Table S1 (Supplementary Material) and Figure 1 presents the graphical representations of the results where solid lines in each graph indicate the limit for each parameter that allows estimating the authenticity of honey. Fructose and glucose were the main sugars found in the samples. Although the concentration of both sugars is variable depending on the origin of honey, in general, fructose is expected to occur in a greater proportion than glucose [41,42]. In fact, Table S1 shows that in more than 50% of the samples fructose is the most abundant sugar. The content of reducing sugars corresponds to the sum of glucose and fructose and, according to the Ecuadorian Technical Standard NTE INEN 1572, the content of reducing sugars should not be less than 65% for authentic honey. However, there are 4 samples (1, 2, 8 and 18) that showed lower than the mentioned minimum content. Low contents of reducing sugars in samples 1, 2 and 18 are directly related to the high content of sucrose in these samples. The sucrose could either come from a digestive issue with the bees [43] or adulteration of the product.

The content of sucrose should not exceed 5% according to the mentioned Standard; in Figure 1b can be observed that two samples (1 and 2) show an abnormally high content of sucrose, while other five samples (10, 13, 14, 18 and 20) display a slight excess above the limit (in a range from 5.2–6.2%). The determination of this parameter is important since can provide hints of adulteration or bad practices in honey production such as artificial bee feeding using some type of sugar syrup, the addition of adulterants directly into the honey after being harvested or an inadequate ripening of the recollected honey [6,41,42]. Also, sugars content in honey is botanical-feed dependent which is contemplated in the Codex Standard, for example, for sucrose is allow up to 10% *w*/*w* if the bees feed with alfalfa (*Medicago sativa*), *Citrus* spp. and others. Those crops were mentioned in the labels of the honey samples of this study and could explain the closer deviation from the 5% of the sucrose limit content in several samples. 

The moisture content (%) of the different samples is in the range of 10.4% to 19.0% (Figure 1c) which are values below the limit established by both the Codex Alimentarius and the Ecuadorian Technical Standard. The moisture content is a factor that may influence the storage ability of honey; very high moisture content can affect the stability of the product and effects on flavour could eventually be noticed [44] due to the growth of osmotolerant yeasts and subsequent formation of ethyl alcohol and carbon dioxide [42,45,46]. The electrical conductivity of honey is related to the content of mineral salts, organic acids as well as proteins and its value mainly varies according to the botanical origin or degree of dilution. The Codex Alimentarius requires honey to have an electrical conductivity no greater than 0.8 mS cm^−1^: The samples subject of the study showed electrical conductivity values within the range of 0.020 to 1.149 mS cm^−1^ (Figure 1d), similar to other reported values [47,48]. Two samples (8 and 14) exceed the allowed limits and could be attributed to the origin of honey since honeydew honeys usually have a high electrical conductivity [49]. However, the adulteration of honey cannot be ruled out because the addition of syrups prepared with water with high electrical conductivity could produce the same effect.

### 3.2. Raman and Infrared Spectra

**Figure 2:**
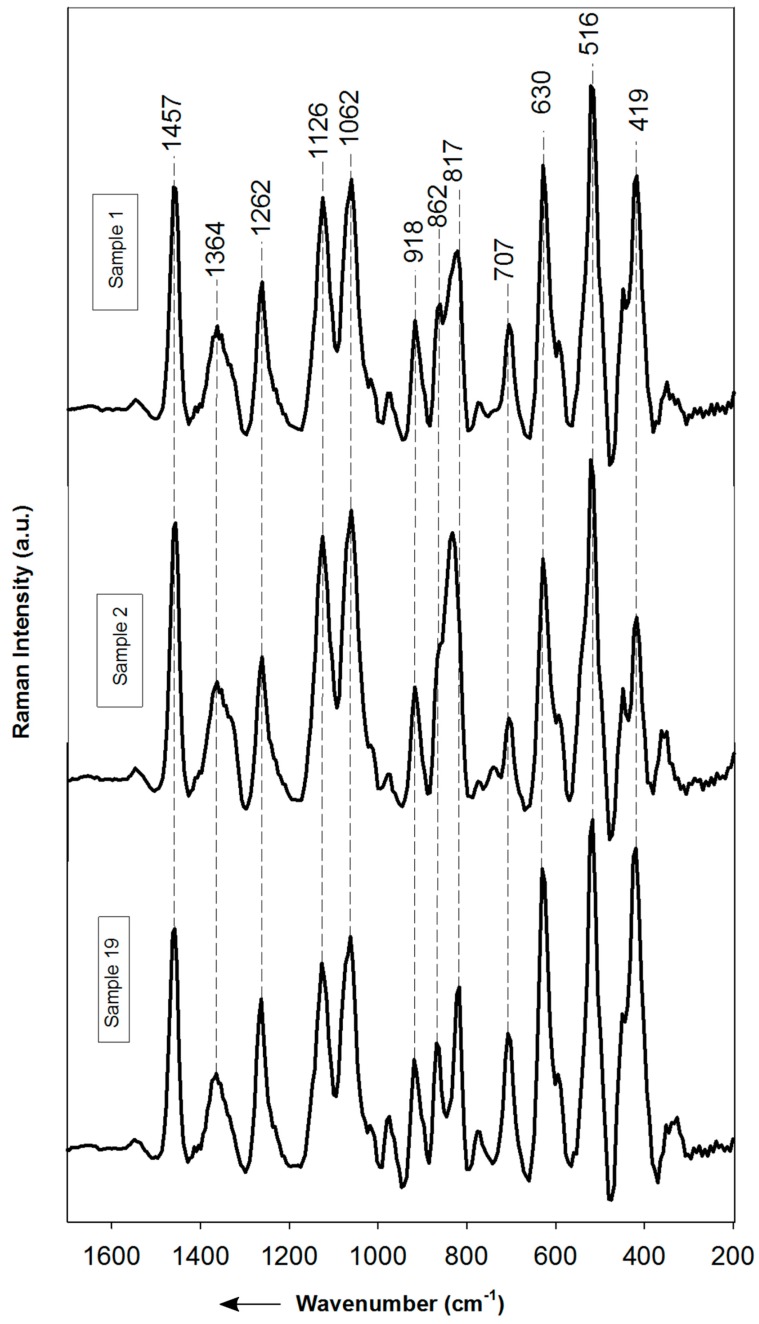
Average Raman spectra of selected honey samples. Samples 1 and 2 show unusual contents of glucose, fructose and sucrose, while sample 19 (considered a sample of pure honey) comply with the contents of the three sugars according to the National Standard.

Selected average Raman spectra of honey samples are presented in Figure 2. Additionally, the complete set of average Raman and IR Spectra of the samples are shown in Figures S1 and S2 (Supplementary Information), respectively. It is well known that Raman spectroscopy is a non-destructive method and often samples do not need special preparation steps [22]. Also, Raman measurements can be performed through transparent glass vials or plastic packages. The Raman spectra can be considered as chemical fingerprints of the materials [50] and are closely related to their composition. In this sense, honey is mainly composed of three sugars: fructose, glucose and sucrose. Sucrose is a disaccharide that consists of one molecule of fructose and a glucose and therefore shows similar Raman features than free fructose and glucose. However, certain characteristic vibrational modes are useful to differentiate the sugars based on structural changes [51]. Glucose has its dominant skeletal vibrational modes δ(C–C–C), δ(C–C–O), δ(C–O) and τ(C–C) reported in the range of 200–500 cm^−1^ [51,52]. The bands at 415 and 437 cm^−1^ are attributed to the δ(C2–C1–O1) bending vibration of α- and β- glucose respectively [52]. The band at 523 cm^−1^ was assigned to the skeletal vibration of glucose [51]. For fructose vibrational spectra it has been proposed, the bands at 874 and 826 cm^−1^ correspond to the C-C stretching modes in the furanoid and pyranoid rings respectively [53]. The strong bands at 419 and 631 cm^−1^ are related with the δ(C-C-O) ring vibration in the pyranoid ring and to a ring deformation respectively [51,52]. Ilaslan, Boyaci and Topcu [51] assigned the bands in the range 820 to 950 cm^−1^, at ν(C-O), δ(C-C-H), ν(C-C) and δ(C-C-O) vibrations of glucose and fructose. On the other hand, sucrose has an intense band at 836 cm^−1^ which corresponds to the ν(C-C) stretching mode [54] and the band at 460 cm^−1^ in the Raman spectra of sucrose corresponds to a strong skeletal vibration [55]. Absorptions at 744 and 800 cm^−1^ might be related to the ν(C-C) vibration of fructopyranose and fructofuranose, respectively [52]. The α-glycosidic bond of C1 on the glucosyl subunit corresponds to the band at 544 cm^−1^ [51,52]. The peak at 1127 cm^−1^ was previously assigned to the C-OH deformation [51], while a strong band at 1368 cm^−1^ is assigned to the CH and OH bending mode of the sucrose [55,56].

Raman spectra of honey samples from Pichincha and Loja provinces show bands at 326, 338, 419, 516, 630, 707, 817, 862, 918, 1062 and 1126 cm^−1^, which can be attributed to the sugars expected to occur in honey (glucose, fructose and sucrose). Figure 2 shows the Raman spectra of two allegedly adulterated samples (1 and 2) and a honey sample (19) considered “pure” because complies with the National Standard requirements. The Raman spectrum of Sample 19 shows well-defined bands at 817 and 862 cm^−1^ which are assigned to the fructose C-C stretching modes. For Samples 1 and 2, these bands seem to be overlapped with the strong absorptions at 822 and 834 cm^−1^ attributable to sucrose. It is important to remark that bands at 822 and 834 cm^−1^ were observed only in the spectra of Samples 1 and 2. The strong band at 630 cm^−1^ may be assigned to a ring deformation of the fructose [51] and the band at 516 cm^−1^ could be related to δ(C-2-C-1-O-1) β bending mode of glucose [54]. The bands at 415 and 437 cm^−1^ (glucose) and at 419 cm^−1^ (fructose) are overlapped, as was also reported earlier by Özbalci et al. [52]. In addition, Goodacre et al. [55] reported similar bands to those observed in this work at 1126, 1262, 1364 and 1457 cm^−1^ which are assigned to C-OH deformation of the glucose and sucrose, C-O-C cyclic alkyl ethers of the fructose, CH and OH bending modes of the glucose and sucrose and CH_2_ bending mode of the fructose, respectively.

**Figure 3:**
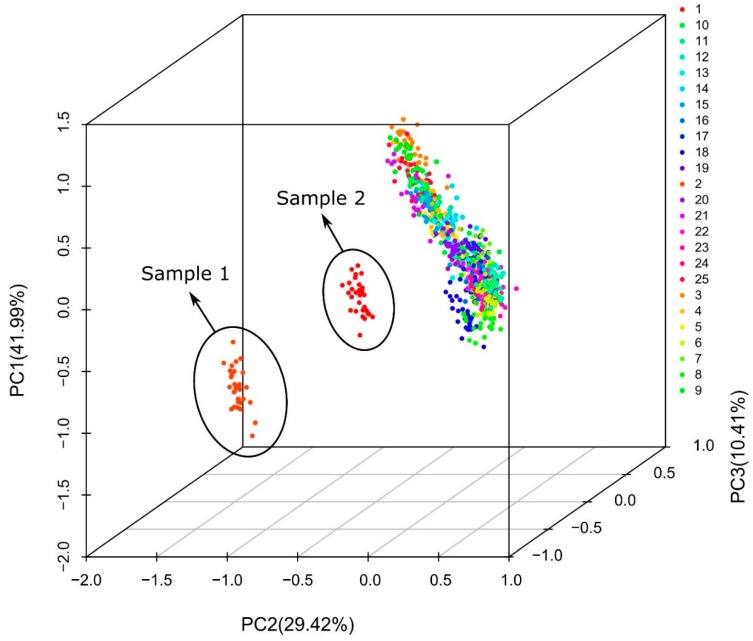
Principal component analysis (PCA) plot for the Raman spectra of 25 honey samples. Sample 1 (23.3% sucrose, 53.3% reducing sugars) and Sample 2 (38.91% sucrose, 38.42% reducing sugars) are considered allegedly adulterated honeys. Samples 8, 10, 13, 14, 18 and 20 show slight variations in the content of sucrose or reducing sugars respect to values established in the National Standard. The other samples comply with the Standard (≤5% sucrose, ≥65% reducing sugars).

A visual inspection of the Raman spectra of Figure 2 allows observing differences between Sample 19 (considered pure honey) and Samples 1 and 2 (allegedly adulterated honey samples). However, the application of the Principal Component Analysis (PCA) can provide objective discrimination of them. The PCA results applied to the Raman spectra of the 25 honey samples are present in Figure 3. As is shown in the graph, the Raman measurements of samples 1 and 2 are different from the other 23 samples. Three well-defined groups can be separated; Samples 1 and 2 are considered as allegedly adulterated honeys because they show low percentages of reducing sugars as well as abnormally high contents of sucrose. Although there are some other samples that exceed the limits of sucrose, the excesses are minimal and these samples are kept within the group that meets the Standard requirements. The obtained results shows that identification of potential adulterated honey is possible using Raman spectroscopy, however, identification of substandard honey can be challenging. 

**Figure 4:**
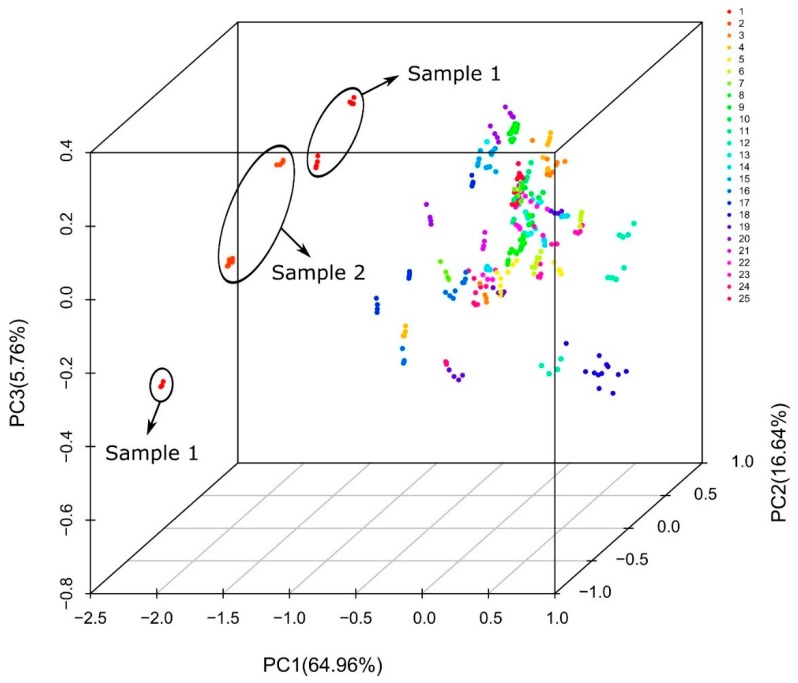
PCA plot for the infrared spectra of 25 honey samples. Sample 1 (23.3% sucrose, 53.3% reducing sugars) and Sample 2 (38.91% sucrose, 38.42% reducing sugars) are considered allegedly adulterated honeys. Samples 8, 10, 13, 14, 18 and 20 show slight variations in the content of sucrose or reducing sugars respect to values established in the National Standard. The other samples comply with the Standard (≤5% sucrose, ≥65% reducing sugars).

The application of the PCA to the infrared spectra of the 25 honey samples are shown in the graph of Figure 4. Different clusters for each sample can be observed, however, Samples 1 and 2 are clearly separated from all the other samples. This is in good agreement with the PCA results obtained for the Raman spectra and confirms the applicability of vibrational spectroscopy for the detection of potential adulterated honey samples.

### 3.3. Pesticides Residues

Residues from 34 compounds (between active ingredient and metabolites) were analysed and the results are shown in Table S2 (Supplementary Materials). Only, one sample of the 25 evaluated tested positive for DMF, an amitraz metabolite in a concentration of 20 μg/kg. The European Union established a Maximum Residue Limit (MRL) of 200 μg/kg for amitraz and DMA (another amitraz metabolite) [57]. Also, the U.S. Environmental Protection Agency (EPA) established a MRL (78 FR 17123) of 200 μg/kg for amitraz and its metabolites and degradation products. Therefore, the positive honey sample for amitraz is below the LRMs mentioned above. Calatayud-Vernich et al. [58] and Lambert et al. [59] detected amitraz metabolites in beekeeping matrices, also below the LMRs. Amitraz is an acaricide which is used inside the hives to control varroa parasite, therefore bees are directly exposed [58,60] and this pesticide can finally move to the honey like in one sample of this study.

## 4. Conclusions

From the 25 commercial honey samples collected in the provinces of Pichincha and Loja, eight samples showed certain deviations from the parameters established in the Ecuadorian and Codex Standards. However, two samples (1 and 2) presented the most significant differences, in its content of sucrose and reducing sugars, showing a presumable case of adulteration. In these cases, the consumers are exposed to adulterated products with the subsequent risks even for their health. Fortunately, different strategies could be applied to determine the authenticity such as physicochemical analysis and vibrational spectroscopic techniques (Raman and IR) combined to chemometric methods like Principal Component Analysis (PCA) for better differentiation and interpretation. Spectroscopic methods are especially suitable for this kind of evaluation since it is fast, requires a minimum amount of sample, are non-destructive techniques and, in this particular case, is low cost compared to the monitoring the sugars by liquid chromatography. Regarding, to the presence of pesticides residues, DMF of amitraz was detected although below the LRM levels. However, it is necessary to be careful with the use of this pesticide as treatment of mites in honey hives. Nevertheless, considering the results obtained in this work it seems that in Ecuador broad monitoring of the quality and authenticity of honey is required.

## Supplementary Materials

The following are available online at https://www.mdpi.com/2304-8158/8/3/105/s1, Table S1: Physic—Chemical Analysis Results, Table S2: Pesticides Residues Results, Figure S1: Average Raman spectra of the 25 samples of honey. Spectra of samples 1 and 2 are drawn in red, Figure S2: Average infrared spectra of the 25 samples of honey. Spectra of samples 1 and 2 are drawn in red.
