# Supplementary material for: Exploratory Monitoring of the Quality and Authenticity of Commercial Honey in Ecuador
Source: Foods. 2019 Mar 20;8(3):105. doi: 10.3390/foods8030105 (PMC6462972; doi:10.3390/foods8030105)
Supplement: Supplementary file 1 [file foods-08-00105-s001.pdf]

SUPPLEMENTARY MATERIAL

Exploratory Monitoring of the Quality and Authenticity of Commercial Honey in Ecuador

Lorena Salvador [1], Michelle Guijarro [1], Daniela Rubio [1], Bolívar Aucatoma [2], Tanya Guillén [2], Paul Vargas Jentzsch [3], Valerian Ciobotă [4], Linda Stolker [5], Sonia Ulic [6], Luis Vásquez [7], Patricia Garrido [1], Juan Bravo [1], Luis Ramos Guerrero [1,*]

[1] Centro de Investigación de Alimentos, CIAL, Universidad UTE, Quito, Ecuador.
[2] Centro de Investigación de la Caña de Azúcar, CINCAE, El Triunfo, Ecuador.
[3] Departamento de Ciencias Nucleares, Facultad de Ingeniería Química y Agroindustria, Escuela Politécnica Nacional, EC170525 Quito, Ecuador
[4] Rigaku Analytical Devices, Jena, Germany
[5] Wageningen University & Research Akkermaalsbos 2, 6708 WB Wageningen, The Netherlands.
[6] CEQUINOR (UNLP-CONICET), Universidad Nacional de La Plata, La Plata, Argentina.
[7] Facultad de Ciencias de la Seguridad y Gestión de Riesgos, Universidad Internacional del Ecuador, Quito, Ecuador

[*] Correspondence: luis.ramos@ute.edu.ec, Cel. +593 980548444

Table S1. Physico – Chemical Analysis Results

Sample	Glucose (%)	Fructose (%)	Reducing Sugar[a] (%F+G)	Sucrose[b] (%)	Moisture[c] (%)	Cond[d] (mS/cm)
1	27.26	26.04	53.30	23.30	12.8	0.243
2	19.72	18.70	38.42	38.91	12.7	0.273
3	39.34	34.62	73.96	1.93	12.5	0.228
4	35.93	38.19	74.12	3.57	11.6	0.190
5	32.29	40.61	72.90	4.45	13.3	0.459
6	29.57	41.06	70.63	2.71	11.5	0.572
7	35.97	39.22	75.19	1.25	12.1	0.415
8	28.20	35.69	63.89	4.02	12.0	1.149
9	39.24	34.80	74.04	3.04	11.8	0.168
10	33.37	36.96	70.33	5.43	12.9	0.616
11	35.67	38.86	74.53	4.27	11.6	0.542
12	27.13	44.67	71.80	1.62	14.7	0.316
13	31.59	37.50	69.09	6.18	12.2	0.454
14	30.66	36.87	67.53	5.52	13.2	0.964
15	37.31	36.36	73.67	4.39	10.6	0.428
16	31.68	38.23	69.91	3.19	10.9	0.604
17	33.73	39.55	73.28	3.90	9.6	0.656
18	26.54	30.63	57.16	5.20	19.0	0.209
19	36.56	38.03	74.59	2.18	13.5	0.386
20	37.59	34.65	72.23	5.79	11.2	0.249
21	34.30	36.18	70.48	2.21	12.3	0.023
22	32.99	37.70	70.68	3.06	12.3	0.022
23	30.83	39.20	70.03	2.80	13.6	0.021
24	31.30	37.19	68.49	2.87	12.9	0.020
25	37.40	37.51	74.91	4.90	10.4	0.020

[a]Reducing sugars (%): ≥65
[b]Sucrose (%): ≤5
[c]Moisture (%):≤20
[d]Cond mS/m: ≤0.8

Table S2. Pesticides Residues Results

PESTICIDES	LOQ* (µg/kg)	SAMPLES																								
		**1**	**2**	**3**	**4**	**5**	**6**	**7**	**8**	**9**	**10**	**11**	**12**	**13**	**14**	**15**	**16**	**17**	**18**	**19**	**20**	**21**	**22**	**23**	**24**	**25**
Abamectin	10	<	<	<	<	<	<	<	<	<	<	<	<	<	<	<	<	<	<	<	<	<	<	<	<	<
Acetamiprid	1	<	<	<	<	<	<	<	<	<	<	<	<	<	<	<	<	<	<	<	<	<	<	<	<	<
DMA (Amitraz metabolite)	25	<	<	<	<	<	<	<	<	<	<	<	<	<	<	<	<	<	<	<	<	<	<	<	<	<
DMF (Amitraz metabolite)	5	<	<	<	<	<	<	<	<	<	<	<	<	<	<	<	20	<	<	<	<	<	<	<	<	<
DMPF (Amitraz metabolite)	5	<	<	<	<	<	<	<	<	<	<	<	<	<	<	<	<	<	<	<	<	<	<	<	<	<
Bendiocarb	1	<	<	<	<	<	<	<	<	<	<	<	<	<	<	<	<	<	<	<	<	<	<	<	<	<
Bifenazate	1	<	<	<	<	<	<	<	<	<	<	<	<	<	<	<	<	<	<	<	<	<	<	<	<	<
Chlorpyriphos	1	<	<	<	<	<	<	<	<	<	<	<	<	<	<	<	<	<	<	<	<	<	<	<	<	<
Coumaphos	2	<	<	<	<	<	<	<	<	<	<	<	<	<	<	<	<	<	<	<	<	<	<	<	<	<
Cypermethrin	5	<	<	<	<	<	<	<	<	<	<	<	<	<	<	<	<	<	<	<	<	<	<	<	<	<
Deltametrin	5	<	<	<	<	<	<	<	<	<	<	<	<	<	<	<	<	<	<	<	<	<	<	<	<	<
Dimethoate	1	<	<	<	<	<	<	<	<	<	<	<	<	<	<	<	<	<	<	<	<	<	<	<	<	<
Omethoate (Also dimethoate metabolite)	1	<	<	<	<	<	<	<	<	<	<	<	<	<	<	<	<	<	<	<	<	<	<	<	<	<
Emamectin	2	<	<	<	<	<	<	<	<	<	<	<	<	<	<	<	<	<	<	<	<	<	<	<	<	<
Fipronil	0.5	<	<	<	<	<	<	<	<	<	<	<	<	<	<	<	<	<	<	<	<	<	<	<	<	<
Fipronil-sulfone (MB46136) (Fipronil metabolite)	0.5	<	<	<	<	<	<	<	<	<	<	<	<	<	<	<	<	<	<	<	<	<	<	<	<	<

Fipronil-carboxamide<br>(Fipronil metabolite)	0.5	<	<	<	<	<	<	<	<	<	<	<	<	<	<	<	<	<	<	<	<	<	<	<	<	<
Fipronil-desulfinyl<br>(Fipronil metabolite)	0.5	<	<	<	<	<	<	<	<	<	<	<	<	<	<	<	<	<	<	<	<	<	<	<	<	<
Fipronil-sulfide<br>(Fipronil metabolite)	0.5	<	<	<	<	<	<	<	<	<	<	<	<	<	<	<	<	<	<	<	<	<	<	<	<	<
Fluvalinate tau	5	<	<	<	<	<	<	<	<	<	<	<	<	<	<	<	<	<	<	<	<	<	<	<	<	<
Imidacloprid	5	<	<	<	<	<	<	<	<	<	<	<	<	<	<	<	<	<	<	<	<	<	<	<	<	<
Imidacloprid 5-Hydroxy<br>(imidacloprid metabolite)	5	<	<	<	<	<	<	<	<	<	<	<	<	<	<	<	<	<	<	<	<	<	<	<	<	<
Imidacloprid desnitro<br>(imidacloprid metabolite)	5	<	<	<	<	<	<	<	<	<	<	<	<	<	<	<	<	<	<	<	<	<	<	<	<	<
Imidacloprid desnitro olefin<br>(imidacloprid metabolite)	5	<	<	<	<	<	<	<	<	<	<	<	<	<	<	<	<	<	<	<	<	<	<	<	<	<
Imidacloprid olefin<br>(imidacloprid metabolite)	50	<	<	<	<	<	<	<	<	<	<	<	<	<	<	<	<	<	<	<	<	<	<	<	<	<
Imidacloprid urea<br>(imidacloprid metabolite)	5	<	<	<	<	<	<	<	<	<	<	<	<	<	<	<	<	<	<	<	<	<	<	<	<	<
6-Chloronicotinic acid<br>(imidacloprid metabolite)	100	<	<	<	<	<	<	<	<	<	<	<	<	<	<	<	<	<	<	<	<	<	<	<	<	<
Indoxacarb	2	<	<	<	<	<	<	<	<	<	<	<	<	<	<	<	<	<	<	<	<	<	<	<	<	<
Propiconazole	5	<	<	<	<	<	<	<	<	<	<	<	<	<	<	<	<	<	<	<	<	<	<	<	<	<
Pyridaben	1	<	<	<	<	<	<	<	<	<	<	<	<	<	<	<	<	<	<	<	<	<	<	<	<	<
Thiacloprid	1	<	<	<	<	<	<	<	<	<	<	<	<	<	<	<	<	<	<	<	<	<	<	<	<	<
Clothianidin<br>(Also thiamethoxam metabolite)	2	<	<	<	<	<	<	<	<	<	<	<	<	<	<	<	<	<	<	<	<	<	<	<	<	<

Thiamethoxam	2	<	<	<	<	<	<	<	<	<	<	<	<	<	<	<	<	<	<	<	<	<	<	<	<	<
Triflumizole	1	<	<	<	<	<	<	<	<	<	<	<	<	<	<	<	<	<	<	<	<	<	<	<	<	<

*Limit of Quantitation

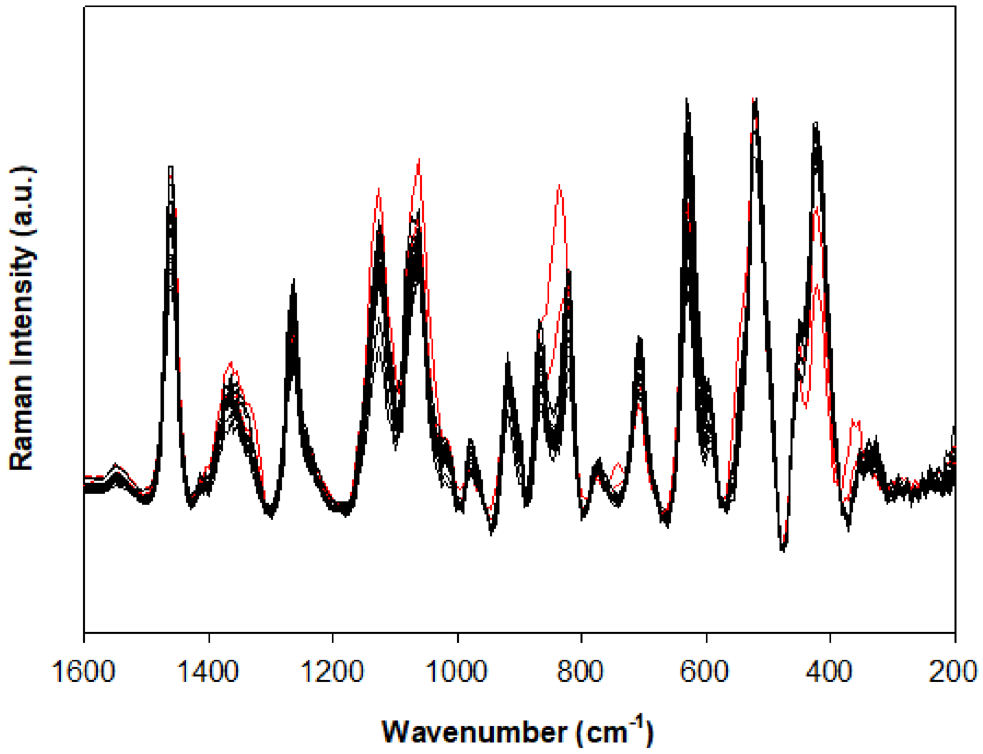

Figure S1. Average Raman spectra of the 25 samples of honey. Spectra of samples 1 and 2 are drawn in red.

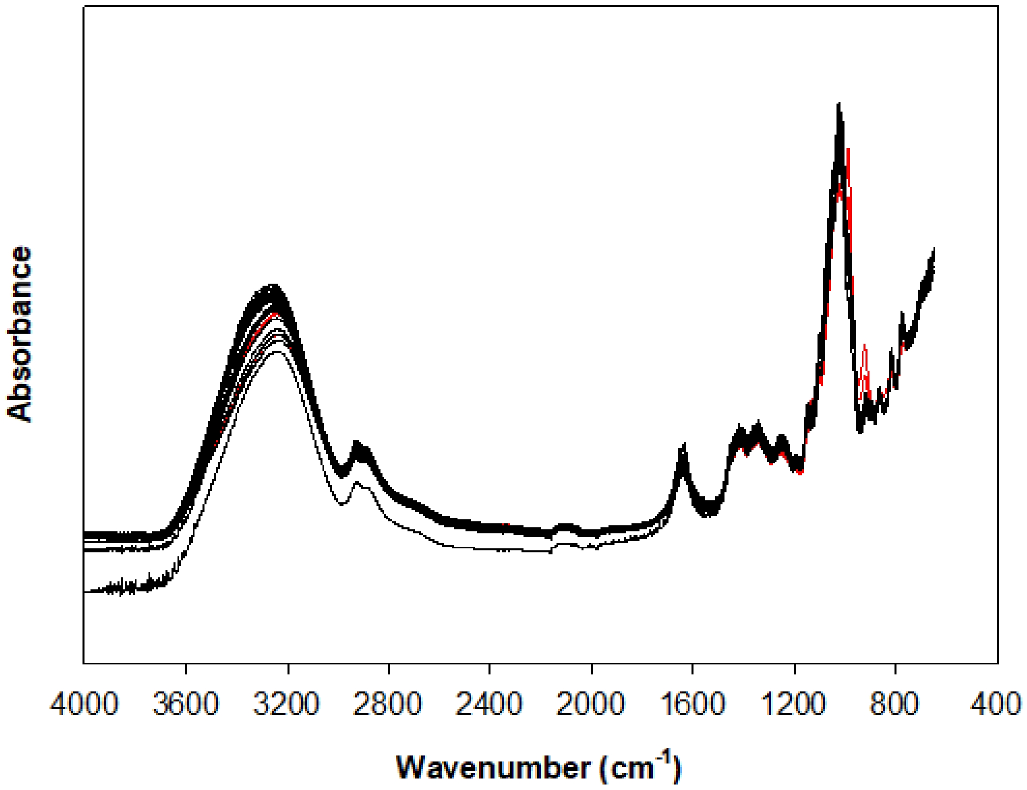

Figure S2. Average infrared spectra of the 25 samples of honey. Spectra of samples 1 and 2 are drawn in red.
